# Crossmodal Integration of Conspecific Vocalizations in Rhesus Macaques

**DOI:** 10.1371/journal.pone.0081825

**Published:** 2013-11-13

**Authors:** Christa Payne, Jocelyne Bachevalier

**Affiliations:** 1 Department of Psychology, Emory University, Atlanta, Georgia, United States of America; 2 Division of Developmental and Cognitive Neuroscience, Yerkes National Primate Research Center, Atlanta, Georgia, United States of America; CNR, Italy

## Abstract

Crossmodal integration of audio/visual information is vital for recognition, interpretation and appropriate reaction to social signals. Here we examined how rhesus macaques process bimodal species-specific vocalizations by eye tracking, using an unconstrained preferential looking paradigm. Six adult rhesus monkeys (3M, 3F) were presented two side-by-side videos of unknown male conspecifics emitting different vocalizations, accompanied by the audio signal corresponding to one of the videos. The percentage of time animals looked to each video was used to assess crossmodal integration ability and the percentages of time spent looking at each of the six *a priori* ROIs (eyes, mouth, and rest of each video) were used to characterize scanning patterns. Animals looked more to the congruent video, confirming reports that rhesus monkeys spontaneously integrate conspecific vocalizations. Scanning patterns showed that monkeys preferentially attended to the eyes and mouth of the stimuli, with subtle differences between males and females such that females showed a tendency to differentiate the eye and mouth regions more than males. These results were similar to studies in humans indicating that when asked to assess emotion-related aspects of visual speech, people preferentially attend to the eyes. Thus, the tendency for female monkeys to show a greater differentiation between the eye and mouth regions than males may indicate that female monkeys were slightly more sensitive to the socio-emotional content of complex signals than male monkeys. The current results emphasize the importance of considering both the sex of the observer and individual variability in passive viewing behavior in nonhuman primate research.

## Introduction

Successful integration into complex social environments requires humans and nonhuman primates to recognize, manipulate, and behave according to the immediate social context. Key elements of this task are building representations of relations between self and others, and flexibly using these representations to guide social behavior [1,2]. This set of skills relies upon the ability to distinguish and interpret social cues that are often broadcast over multiple sensory modalities. Hence, crossmodal integration has become a crucial component of social success in primates. 

The remarkable behavioral [3-6] similarities between humans and nonhuman primates include the use of species-specific facial expressions and vocalization [7-9]. For both species, decoding the specific “message” of a social display relies on crossmodal integration. The rhesus communicative system is comprised of a small repertoire of relatively fixed calls characterized with distinct facial expressions, postures, and gestures and associated with particular social contexts. This repertoire has been successfully used to explore the evolutionary basis and neural mechanisms of visual speech perception (reviewed by [10]). 

Recent studies have demonstrated that rhesus macaques spontaneously recognize the correspondence between facial and vocal expressions [11]. When pairs of videos depicting two different conspecific vocalizations (i.e., coo and threat) are presented simultaneously with the auditory track matching one of the videos, rhesus macaques look longer to the congruent stimulus video. This is interpreted as spontaneous integration of the auditory and visual components of the stimuli. This paradigm, however, does not rule out the possibility that monkeys merely rely upon the temporal coincidence of facial movements with the onset of the vocal track. A subsequent electrophysiological experiment using the same videos presented sequentially and including a non-biological, mechanical control that mimicked the mouth movements of the videos (in space and time) indicates that integration of the bimodal vocalizations is not dependent upon temporal coincidence [12]. However, given that the videos in the latter experiment were presented individually, the possibility remains that the preference for congruence observed in the preferential viewing paradigm is attributable to the mechanical or temporal coincidence of the auditory and visual components of the stimulus videos. The mechanisms underlying this spontaneous preference for congruence have yet to be systematically explored; and little is known about the visual scanning strategies used by monkeys during crossmodal integration. It has been demonstrated that human subjects modify their scanning strategies of audiovisual stimuli based on the information they are instructed to extract and the efficacy of the social signals [13-16]. It has also been suggested that men and women are differentially sensitive to the emotional content of audiovisual social communication [17], which may manifest as sexual dimorphic scanning strategies.

To date, the only investigation to monitor how monkeys look at socially salient bimodal stimuli was designed to explore the evolutionary basis for humans’ use of facial cues to enhance speech comprehension [18]. This report highlighted the importance of the eye region to rhesus monkeys, but did not directly identify the facial cues needed to support a preference for congruence. Nor did this report assess sex differences in the way male and female rhesus macaques process socio-emotional stimuli. Accordingly, the goals of the present investigation were to assess integration ability in surrogate nursery-reared male and female rhesus macaques using a preferential viewing paradigm; determine whether spontaneous integration ability is solely dependent upon temporal or mechanical coincidence of the auditory and visual components of species-typical vocalizations using an ethologically relevant mechanical control; and characterize the scanning strategies during the preferential viewing paradigm to determine what features the male and female rhesus macaques use to process the stimuli using eye-tracking technology. 

## Method

### Ethics Statement

All procedures were approved by the Animal Care and Use Committee of the University of Texas Health Science Center at Houston in Houston, TX and of Emory University in Atlanta, GA and carried out in accordance with the National Institutes of Health Guide for the Care and Use of Laboratory Animals. Power analyses were completed to determine that a minimum of 5 trials were required to detect large effects at 80% power in a cohort of monkeys with 3 males and 3 females.

### Subjects

Six adult rhesus monkeys (*Macaca mulatta*) aged 4-6 years (3 males, 3 females) were used in this investigation. Animals were surrogate-peer reared in a socially enriched environment that promoted species-specific social skills and alleviated psychological stress [19-21]. Surrogate-peer rearing involved individual housing in size-appropriate wire cages that allowed physical contact with animals in neighboring cage(s), as well as visual, auditory, and olfactory contact with all other infants in the nursery. Each infant was provided a synthetic plush surrogate and cotton towels for contact comfort. The infants received daily social interaction with age- and sex-matched peers as well as with human caregivers, and had repeated assessments of memory, emotional reactivity, social behavior, and reward appraisal throughout their lives.  These animals served as sham-operated controls in a program of experiments designed to characterize the functional and neuroanatomical development of hippocampus, amygdala and orbital frontal cortex. Accordingly, they received sham operations at 10-12 days of age, which included small bilateral craniotomies with no penetration of the dura layer (for details, see 19) and underwent multiple magnetic resonance imaging (MRI) scans to assess gross neural development between 2 weeks and 2.5 years of age [22].  All neuroimaging and surgical procedures were performed under deep anesthesia (Isoflurane, 1-2%) and using aseptic procedures. Animals received pre- and post-surgical treatments to minimize risk of infection (Cephazolin, 25 mg/kg, per os) and control swelling (dexamethazone sodium phosphate, 0.4 mg/kg, s.c.). Topical antibiotic ointment (bacitracin-neomycin-polymyxin) was applied daily and acetaminophen (10mg/kg, p.o.) was given four times a day to relieve pain.

### Crossmodal Integration Task

A preferential viewing paradigm similar to that used by Ghazanfar and Logothetis [11] was selected in the present investigation. 

#### Apparatus

Testing was completed in a sound-attenuated room. Monkeys were seated in a primate chair 2-feet from of a 24-inch, flat panel LCD monitor with attached speaker and small eye-tracking camera (60 Hz; ISCAN, Inc.; Woburn, MA). Head movements were gently minimized with a restraint device attached to the primate chair. Ambient white noise was played to further dampen unrelated noises and a curtain concealed all additional equipment.

#### Stimuli

**Figure 1:**
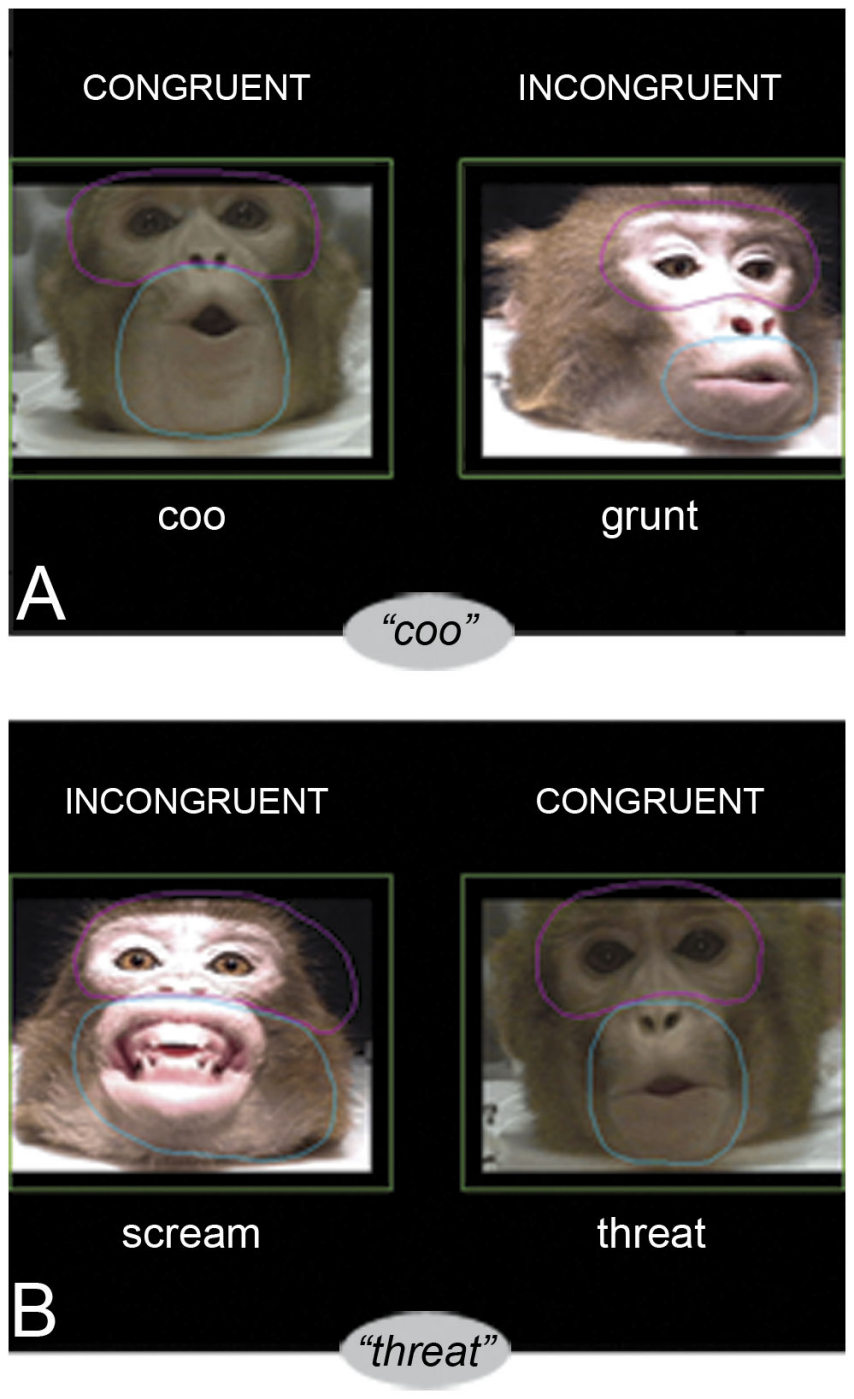
Schematic of Stimulus Presentation with ROIs. Screen shots of coo-grunt (A) and scream-threat (B) pairings with borders of eye and mouth ROIs. In (A), the audible vocalization was a “coo” and in (B), the audible vocalization was a “threat”. ROIs were determined such that the entire region was included throughout the entire video, resulting in slightly extended ROIs in the still representation of the videos. Stimulus sets were comprised of all possible combinations of videos. Labels were not part of stimuli.

Animals were presented two side-by-side digital 2-sec videos of the facial gestures associated with species-typical calls (coo, grunt, scream and threat). The videos were those used by Ghazanfar and Logothetis [11] and depicted two unknown rhesus monkeys (stimulus animals) emitting the vocalizations. One stimulus animal generated the coo and threat vocalizations and the other stimulus animal generated the grunt and scream vocalizations (see Figure 1). Videos were 640 x 480 pixels and spaced apart maximally (200 pixels) on a solid black background. The sound track corresponding with one of the presented facial gestures was heard through the speaker centered beneath the monitor. The auditory and visual components were played in a continuous loop for 10 sec (5 repetitions). Stimulus presentation was controlled using the Presentation software package (Neurobehavioral Systems, Inc; Albany, CA).

#### Task

The auditory component and the left-right position of the two facial gestures were counterbalanced. Stimuli were presented under two different conditions: Synchronized and Desynchronized. The Synchronized condition was used as the standard for integration assessment and were constructed such that the onsets of the auditory and visual components were simultaneous. A total of eight trials in the Synchronized condition were administered across four testing sessions (2 trials/day). The Desynchronized condition was employed to assess whether integration ability relied only upon the mechanical properties of the stimuli (i.e. the coincidence of mouth movements with the auditory component). Trials in the Desynchronized condition were constructed such that the onset of the auditory component was delayed 330 - 430 msec from the onset of the visual component, a delay range that has been shown to disrupt the perception of the stimuli as a single event [23] and resulted in no overlap between the mouth movements and sound. A total of eight trials in the Desynchronized condition were administered across two testing sessions (4 trials/day). 

### Measures

#### Integration Assessment

In a given trial, there was one congruent video (i.e., depicted the facial gestures that matched the audio component) and one incongruent video (i.e., facial gestures did not match the audio track). Crossmodal integration was determined by comparing the percent looking time to each video to the chance level of 50%. Integration of the audio and visual components was inferred when monkeys showed a preference for one of the video clips (i.e., looked significantly more than chance to either the congruent or incongruent stimulus video). Accordingly, an inability to integrate the complex social signals would be demonstrated by monkeys exhibiting equal looking times to each video in the pair. 

#### Scanning Pattern Characterization

Percentages of looking time to *a priori* regions of interest (ROIs) of the videos were recorded. Static ROIs of the eyes and mouth were created with the ISCAN P.O.R. Fixation Analysis software (v1.2, ISCAN, Inc., Figure 1) such that each ROI encapsulated the entire feature of interest throughout the entire 2-sec video. The region of the video not included in either the “eyes” or “mouth” ROI was analyzed as the third ROI labeled “other”. There were six ROIs in each trial: eyes, mouth, and other for each of the two stimulus videos. Scanning patterns were characterized by comparing the amount of time animals spent looking at each ROI, which was calculated from the summation of the fixation durations in a given ROI. A fixation was defined as the eye gaze coordinates remaining within 1° x 1° visual angle for at least 50 msec. Fixations were categorized by ROI using the ISCAN P.O.R. Fixation Analysis Software, and variability in looking time across trials and animals was accounted for by expressing looking to each ROI as a percentage of total looking ((ROI/Total)*100). 

### Statistical Analyses

All measures were normally distributed (Shapiro-Wilk *W* = 0.799-1.000, *p* = 0.112-0.973). Integration abilities were assessed separately for the Synchronized and Desynchronized conditions by comparing the percentages of looking to the congruent stimuli to the chance level of 50% using a one-sample t-test. Repeated measures ANOVA were used to evaluate sex differences and to compare the integration abilities across conditions. Scanning patterns of the ROIs of each stimulus video in a trial were analyzed using repeated measures MANOVA (stimulus video x ROI x sex) with simple interactions and simple comparisons used to conduct planned comparisons of the relative looking to individual ROIs across stimulus video and sex. The assumption of equality of variances was met for all analyses (Levene’s: F(1,4) = 0.007-7.357, *p* = 0.053-0.939) except for two measures in the analysis of the congruent and incongruent stimulus videos across all trials (Levene’s: F(1,4) = 8.952 - 9.336, *p* = 0.038-0.040). Natural log transformations were used to correct for the violations.

## Results

### Overall Integration and Scanning Patterns

#### Integration Assessment

**Figure 2:**
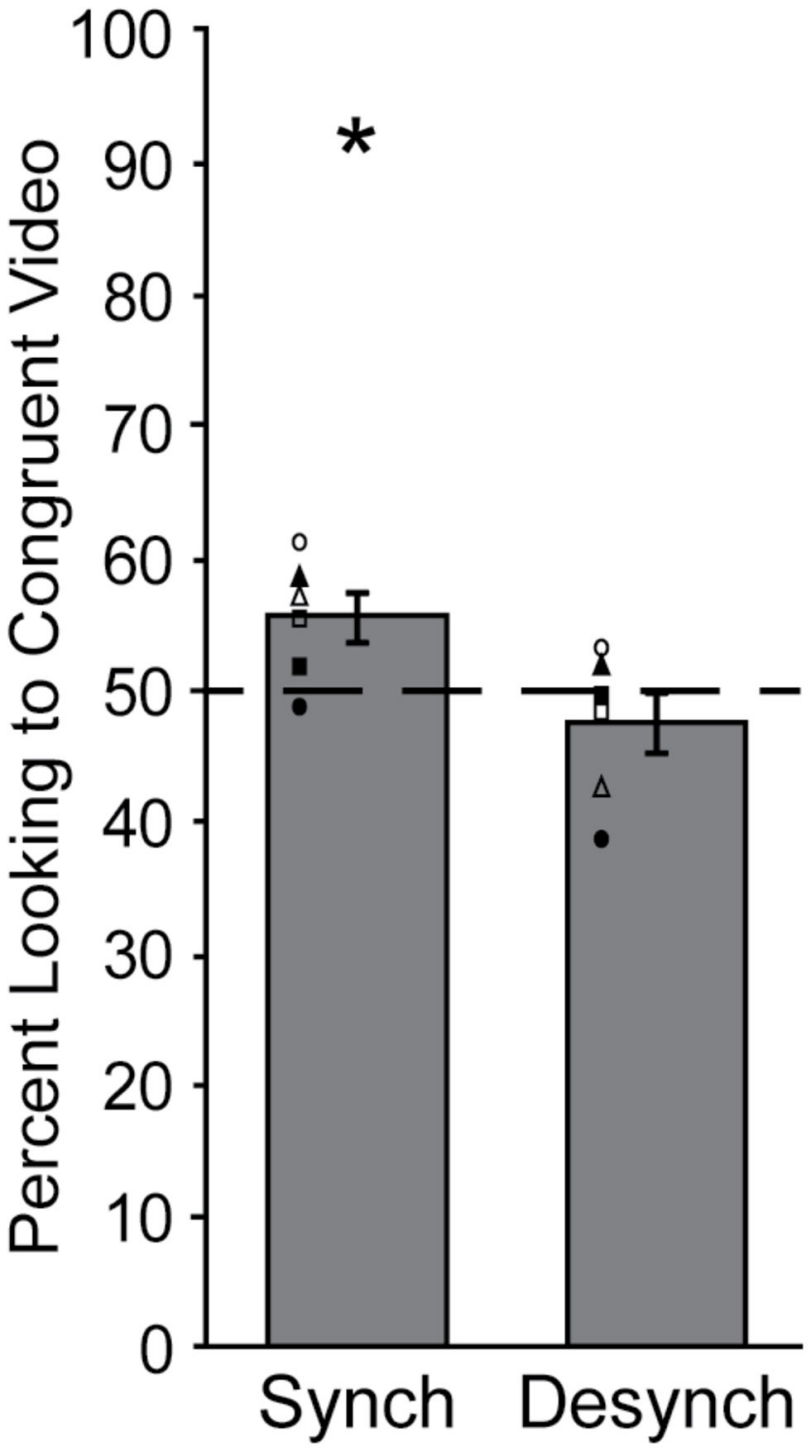
Integration Assessment. Percentages of looking time (± s.e.m.) to the congruent stimulus video in the Synchronized and Desynchronized conditions. The dashed line represents chance level of 50%. Values greater than 50% represent greater looking to the congruent video and values less than 50% represent greater looking to the incongruent video. Symbols represent individual data points for males (filled) and females (open). (*) *p* < 0.05.

In the Synchronized condition (Figure 2), animals exhibited spontaneous integration of complex crossmodal social signals by looking significantly more than chance to the congruent stimulus video (t(5) = 2.941, *p* = 0.032). Qualitatively this effect appears to be driven by the behavior of the females (see Figure 2, open symbols), but this apparent sex difference was not statistically significant (F(1,4) = 2.186, *p* = 0.213). In the Desynchronized condition, animals did not show a preference for congruence (t(5) = -1.115, *p* = 0.316; Figure 2), and males and females did not differ (F(1,4) = 0.060, *p* = 0.819). 

#### Scanning Pattern Characterization

**Figure 3:**
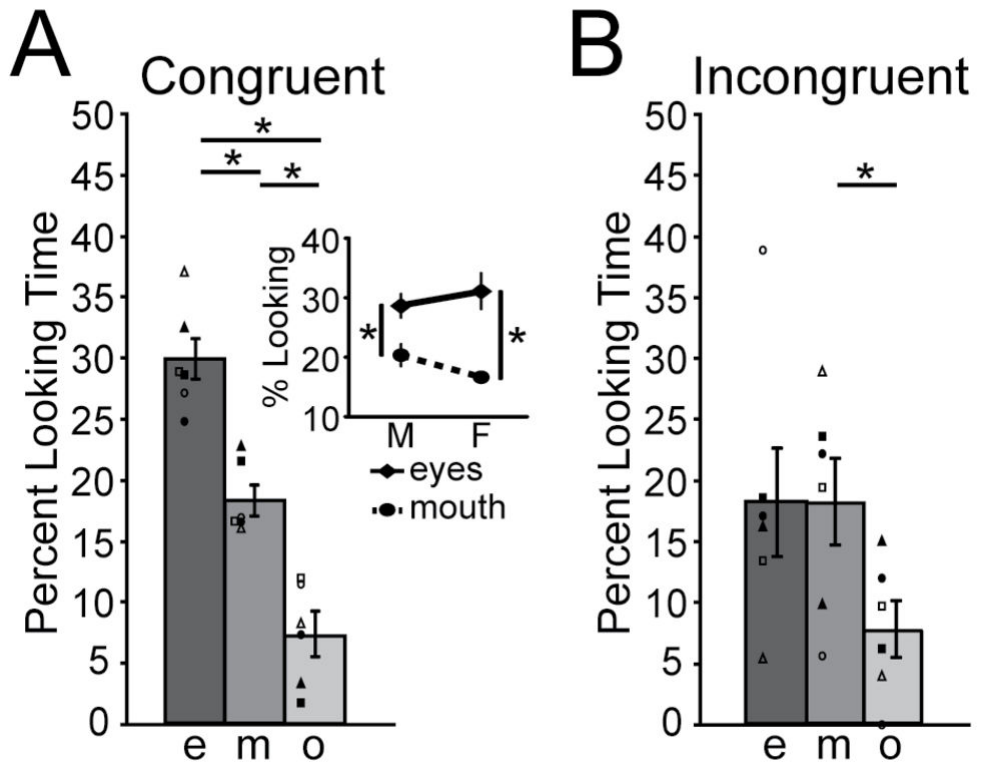
Scanning Patterns. Percentages of looking time (± s.e.m.) to the eyes (e), mouth (m), and other (o) of the congruent stimulus video (A), the incongruent stimulus video (B). Inset in A: Percentages of looking time (± s.e.m.) to the eyes (diamonds/solid line) and mouth (circles/dashed line) of the congruent stimulus video in for males and females. (*) *p* ≤ 0.05.

Figure 3 illustrates notable differences in monkeys’ exploration of the congruent and incongruent stimulus videos in the Synchronized condition. On the congruent stimulus video (Figure 3A), they spent more time looking to the eye region than the mouth region (F(1,4) = 69.115, *p* = 0.001), and looked longer to the eye and mouth regions than to the rest of the video (eyes > other F(1,4) = 45.672, *p* = 0.003; mouth > other F(1,4) = 21.927, *p* = 0.009). There was a weak trend for a sex difference in the relative looking to the eye and mouth regions (ROI x Sex interaction: F(1,4) = 5.262, *p* = 0.084), with females exhibiting a larger differentiation than males (Figure 3A inset). On the incongruent stimulus video (Figure 3B), monkeys spent comparable amounts of time looking at the eyes and either the mouth or rest of the stimulus video (eyes = mouth: F(1,4) = 0.001, *p* = 0.972; eyes = other: F(1,4) = 2.544, *p* = 0.186) but looked more to the mouth than the rest of the stimulus video (mouth > other: F(1,4) = 9.558, *p* = 0.037). No sex differences were observed for the incongruent stimulus video.

There were no interactions of Condition with either ROI (F(2,8) = 0.121, *p* = 0.887) or Stimulus Video and ROI (F(2,8) = 0.086, *p* = 0.918); neither were there interactions between these factors and Sex (Condition x ROI x Sex: (F(2,8) = 0.004, *p* = 0.996; Condition x Stimulus Video x ROI x Sex: F(2,8) = 1.904, *p* = 0.211). This indicates that scanning of trials in the Desynchronized condition did not differ from that of trials in the Synchronized condition. Planned simple effect comparisons of Condition at the individual ROIs confirmed that animals spent comparable proportions of time looking at the eyes (congruent: F(1,4) = 1.550, *p* = 0.281; incongruent: F(1,4) = 2.518, *p* = 0.188), mouth (congruent: F(1,4) = 0.280, *p* = 0.625; incongruent: F(1,4) = 2.406, *p* = 0.196) and rest of the stimulus videos (congruent: F(1,4) = 0.365, *p* = 0.578; incongruent: F(1,4) = 0.014, *p* = 0.911) of the Synchronized and Desynchronized conditions.

## Discussion

The results confirm previous findings that rhesus macaques spontaneously integrate the auditory and visual components of complex social cues emitted by novel conspecific males [11]. They further suggested that these abilities might be influenced by, but perhaps not dependent upon, the mechanical properties of stimuli. Finally, monkeys looked at the eyes of the congruent stimulus video more than other facial cues, with females showing a slightly larger differentiation between eyes and mouth than males. 

### Individual Variability

Before discussing the implications of these results, it is important to acknowledge the impact of individual variability on the current findings. This investigation employed an experimental design that assesses the animals’ *spontaneous* looking behavior. Therefore, unlike more cognitive crossmodal matching tasks that require responders to determine the inter-sensory relatedness of two stimuli in order to receive a reward, there is no *right* or *wrong* video in a preferential viewing paradigm. Inferences were based on where the animals “prefer” to look, which could vary substantially across animals. For example, the female represented by the open triangle demonstrated a preference for congruence in the Synchronized condition but looked more to the incongruent stimulus video in the Desynchronized condition. Assessment of scanning patterns of this animal revealed that it looked most to the eye region of the congruent video, but in the incongruent video, it fixated most on the mouth region. Comparatively, the female represented by the open circle demonstrated a clear preference for the congruent video in the Synchronized condition but looked more equally to the videos in the Desynchronized condition; and this animal’s scanning patterns across the congruent and incongruent videos were strikingly similar to each other, with a strong preference of the eye region in both videos. 

This variability should be considered when interpreting the lack of a preference for congruence in the Desynchronized condition. Studies employing non-social control conditions have previously shown that integration ability does not rely solely on the mechanical properties of the stimuli [12]. This brings to question whether the lack of preference observed in the Desynchronized condition of the current investigation was due to the social complexity of the stimuli. As illustrated in Figure 2, in the Desynchronized condition, two animals looked slightly more towards the congruent video, whereas two animals looked slightly more and two animals looked substantially more towards the incongruent video. The social complexity of the stimuli makes it difficult to interpret how the Desynchronized videos were processed. One reasonable explanation for the variability seen across animals is that different animals focus on different aspects of the stimuli (e.g., social content or mechanical properties). Thus, although the lack of significant preference in the Desynchronized condition could indicate that rhesus macaques relied on the temporal coincidence of the auditory and visual components for integration into a single construct, contradictory previous findings [12] combined with the individual variability and lack of differences in scanning patterns across the Synchronized and Desynchronized conditions observed in the current study suggests that further analysis is needed. 

### Viewing of Eye Regions

Characterization of the scanning patterns indicated that rhesus monkeys attended to the eye regions of the stimulus animals as they evaluated the dynamic, bimodal vocalizations. This interest in the eye region adds to a number of previous studies reporting that both humans and monkeys preferentially investigate the eye regions of conspecifics presented either in static images [24-34] or dynamic, naturalistic videos [18,35-37]. Both humans and rhesus monkeys broadcast important socio-emotional information through their eyes (e.g., their emotional or mental state, social intentions, or focus of their attention), thus attending to the eye region provides the observer with a wealth of socially relevant information [38]. 

Interestingly, males and females exhibited subtle differences in their looking of the eye and mouth regions of the congruent stimulus video, with females showing a slightly greater differentiation between the regions than males. Although differential scanning by males and females has not been empirically investigated in monkeys, previous studies have shown that humans modify their gaze behavior based on the information they intend to extract. Thus, when instructed to focus on emotion-related cues (e.g., prosody) or make social judgments, human subjects look more to the eye region than the mouth region [13,14]. However, when attending to speech-specific aspects of the communication signal (e.g., phonetic details in high levels of ambient noise), they focus significantly more to the mouth region [15,16]. Interestingly, when allowed to passively view videos of vocalizing actors, human subjects also preferentially attend to the eye regions [36,37]. It can thereby be inferred that, during passive viewing, humans preferentially attend to the socio-emotional aspects of the stimuli. By extension, the present findings suggest that monkeys attended to the socio-emotional aspects of the stimuli. The results further suggest that female monkeys may be slightly more sensitive to the socio-emotional content of complex signals than male monkeys. Although further studies are clearly needed to better understand the significance of this sex difference, the data parallel recent findings in humans indicating that women recognize crossmodal emotional expressions of fear and disgust strikingly better than men [17]. 

### Conclusions

Humans and nonhuman primates live in complex social environments where social signals are primarily transmitted via faces and vocalizations. The ability to process audiovisual information is necessary for the recognition of individuals and their emotional states. Rhesus macaques possess the ability to integrate the audio and visual components of species-specific vocalizations, and females may be slightly more attuned to the socio-emotional aspects of complex, species-specific social signals. The current results emphasize that subsequent investigations in nonhuman primates should take into account the sex of the observer, as well as considerable individual variability in passive viewing behavior.

Characterization of these types of naturally occurring behavioral differences in normal subjects and the identification of the neural substrates of those differences are particularly important for research on disorders characterized by deficits in emotional crossmodal integration, such as autism spectrum disorder [39-42], pervasive developmental disorder [43,44]; and schizophrenia [45-47]. Only a few functional neuroimaging studies in humans have begun to identify neuroanatomical correlates of emotional crossmodal integration and have shown greater responses to bimodal emotional expressions (face and voice) than unimodal emotional expressions in the amygdala [48], medial temporal gyrus, anterior fusiform gyrus [49], and posterior superior temporal gyrus ([50]), as well as the thalamus [51]. None have documented sex differences in activation patterns. Although several investigations have empirically demonstrated emotional crossmodal integration abilities in nonhuman primates (e.g. [51-53]), to date, the neural substrates of these abilities in monkeys have yet to be investigated. 
